# Genome-Wide Association Study Revealed Key Genes with the Teat Number in Jishen Black Pigs

**DOI:** 10.3390/vetsci13060537

**Published:** 2026-05-29

**Authors:** Xiaoran Zhang, Hao Sun, Juan Ke, Changyi Chen, Fengyi Dong, Simin Liu, Long Jin, Jing Li, Luyao Bie, Chunyan Bai, Boxing Sun

**Affiliations:** College of Animal Sciences, Jilin University, Changchun 130062, China

**Keywords:** Jishen black pigs, genome-wide association study (GWAS), total teat number (TTN), left teat number (LTN), right teat number (RTN)

## Abstract

As an important reproductive trait, teat number plays a vital role in the pork industry, which is related to the ability of sows to host more piglets. However, the genetic mechanism of the teat number in pigs is still not very clear. We collected phenotype data, including the total teat number (TTN), left teat number (LTN), and right teat number (RTN), from 300 Jishen Black (JSB) sows and performed genotyping using a porcine SNP 50K panel. Most JSB sows exhibited 14, 15, or 16 TTN (259, 86%). Only 41 pigs (14%) had TTN values outside this range. Moreover, the LTN and TTN exhibited approximate frequency distribution in the studied population. Seven was the most common LTN and RTN in JSB sows, accounting for 181 (60%) and 173 (57%), respectively. Additionally, these traits exhibited low-to-medium heritability (0.14–0.22). Using the BLINK and FarmCPU models, three, six, and three key loci were identified in the GWAS of TTN, LTN, and RTN, respectively. Additionally, five key genes were detected to play vital roles in teat number traits, including *ME1*, *SCN8A*, *EVC*, *UBC*, and *PDE4D*. Our research provided efficient molecular markers and new insights into further improvement and understanding for teat number traits in Jishen Black pigs.

## Simple Summary

The total teat number, left teat number, and right teat number are vital economic traits in the pork industry. However, the genetic mechanism of teat number in pigs is still unclear. In this study, we collected phenotype data from 300 Jishen Black sows and performed genotyping using a porcine SNP 50K panel. A total of 11 key SNPs and five candidate genes were detected to be related to pig teat number traits. These findings help researchers better understand these traits and select sows with better reproduction performance.

## 1. Introduction

As key economic indicators, reproduction traits play a vital role in the pork industry, directly associated with the profitability of sows [1]. For domestic sows, reproduction traits comprise a variety of specific components, such as teat number, litter size and litter birth weight [2]. Since the teat number of sows also reflects their body size and lactation capacity, it consequently affects their litter size and litter birth weight [3]. Therefore, deciphering the genetic mechanisms underlying sow teat number traits is of great significance and can provide available molecular markers for the selection and breeding of high-quality breeding pigs and bring higher benefits to the pig industry.

Whereas growth traits typically show high heritability, the teat number in pigs exhibits low-to-medium heritability [4]. Therefore, conventional selection methods are insufficient for improving the teat number [5,6]. Meanwhile, the pig teat number is determined at birth and unaffected by environmental and nutritional conditions [7]. Thus, genomic analysis of this trait is crucial. Recently, researchers found that the heritability estimates for teat number in pigs differ across breeds and among lines within breeds [7]. For instance, in two genomic evaluations of the total teat number in selected Yorkshire pigs, the heritability of TTN was 0.42 for American Yorkshire pigs, whereas it was only 0.17 for the Danish and French lines of Yorkshire pigs [8,9]. Additionally, Zhang et al. reported that the heritability of teat number was 0.19 and 0.34 for American and Canadian Duroc pigs, respectively [10].

Genome-wide association study (GWAS) utilizes population genetic variation information associated with phenotypic information to identify candidate genes related to target traits. It has been widely used to analyze the genetic mechanisms of economic traits in livestock and poultry [11]. Based on GWAS, several important genes associated with teat number in commercial pigs have been identified, such as *VRTN* [3,10,12], *ABCD4* [13,14], and *IGF2* [15]. In addition, researchers have conducted GWAS on many local Chinese pig breeds to explore the genetic mechanisms underlying teat number traits in these populations. For instance, *OLFML2A* was identified as a candidate gene associated with TTN in Meishan and Erhualian pigs [16]. In addition, Ke et al. identified nine candidate genes in Dongliao Black pigs [17]. Additionally, single nucleotide polymorphisms (SNPs) near the *TBX3* and *NTRK2* were supposed to be efficient molecular markers in Qingping pigs and Beijing Black pigs, respectively [18,19].

The Jishen Black (JSB) pig is a newly developed breed with over 20 years of selective breeding, derived from the crossbreeding of Beijing Black pigs and Yorkshire pigs. It is well known for its high meat quality, strong cold tolerance and roughage resistance. In November 2017, the breed was officially certified and granted a breed certificate by the National Livestock and Poultry Genetic Resources Committee. Developed under the auspices of Jilin Jingqishen Organic Agriculture Co., Ltd., JSB pigs are mainly produced in the Jilin Province. The breeding enterprise operates a complete industrial chain and has built a premium Jishen Black pork brand, holding the leading position in China’s high-end pork market, with an annual commercial slaughter of 200,000 heads. There are 3800 basic sows in the core breeding population. Their products are widely marketed in major cities, including Beijing, Shanghai, Guangzhou, and Shenzhen. Despite their economic importance, genetic studies on JSB pigs remain limited, and the genetic mechanisms underlying the teat number variation in this breed are still poorly understood. Therefore, a genome-wide association study is urgently needed to deepen our understanding of teat number traits in JSB pigs, which will further support their genetic improvement and sustainable brand development.

## 2. Materials and Methods

### 2.1. Experimental Animals

Under the instruction of the Institutional Animal Care and Use Committee of Jilin University (IACUC, Changchun, China), 300 Jishen Black sows were housed indoors and provided with adequate feed and water at the breeding farm of Jilin Jingqishen Organic Agriculture Co., Ltd. (Baishan, China).

### 2.2. Phenotype and Samples Collection

The experimental pigs were raised under the same feeding and management conditions in the breeding farm. After farrowing, each sow was housed in a gestation stall. The total teat number, left teat number, and right teat number of each experimental pig were precisely counted and promptly recorded. Additionally, ear tissue samples were collected from 300 JSB sows using tissue scissors and immediately stored in a centrifuge tube containing 75% alcohol solution.

### 2.3. Genotyping and Quality Control

Genomic DNA was extracted strictly according to the instruction of the FastPure Cell/Tissue DNA Isolation Mini Kit-BOX 1 (DC102-02, Vazyme, Nanjing, China), after which we detected the quality of isolated DNA using the NanoDrop 2000 (Thermo Scientific, Shanghai, China). Only isolated DNA meeting the following criteria was considered qualified: (1) the A260/A280 ratio was between 1.7 and 2.1; (2) the concentration was higher than 50 ng/μL. Subsequently, all qualified samples were genotyped using the GenoBaits Porcine SNP 50K Panel, which contains 52,491 SNPs (MolBreeding, Shijiazhuang, China). Additionally, the SNPs were filtered out using VCFtools software (version 0.1.17) [20] in accordance with these criteria: (1) Only autosomal SNPs are retained. (2) Keep SNPs with a call rate higher than 90%. (3) Remove SNPs with a minor allele frequency (MAF) less than 1%. Finally, a total of 46,742 SNPs were retained and imputed using the beagle software (version 5.5) [21] for further analyses.

### 2.4. Estimation of the Breeding Values and Genetic Variance

The HIBLUP software (version 1.6.0) [22] was used to estimate the breeding values and genetic variance with a single-trait model. Default parameters were chosen to run the estimation. The single-trait model used in this study was defined as below:

y=Xb+Za+e

where y represents the vector of the phenotype of TTN, LTN or RTN, and b is the vector of fixed effect (contemporary group); a is the vector of additive genetic effects (breeding values); e is the vector of residual effects; X and Z are the correlation matrices corresponding to b and a, respectively. The parameters a and e both follow normal distributions: $a\sim N\;(0,G\sigma_{a}^{2})$ and $e\sim N\;(0,I\sigma_{e}^{2})$. In these formulas, G and I represent the vectors of the kinship matrix and identify matrix, respectively, $\sigma_{a}^{2}$ is the genetic variance, and $\sigma_{e}^{2}$ is the residual variance. Subsequently, the heritability ($h^{2}$) of teat number traits was calculated by the formula below:

$$h^{2}\;=\;\sigma_{a}^{2}/\;(\sigma_{a}^{2}\;+\;\sigma_{e}^{2})$$

### 2.5. Genome-Wide Association Studies

Following the genetic variance estimation, genome-wide association studies were conducted using the GAPIT software (version 3.5) [23] with the estimated breeding values. The Bayesian-information and Linkage-disequilibrium Iterated Conditional Key (BLINK) model and the Fixed and random model Circulating Probability Unification (FarmCPU) model implemented in the software were chosen to analyze each teat number trait with the first three principal components as covariates, respectively. Additionally, the genome-wide significant threshold was set as 5.971 using the Bonferroni correction method (calculated as $-\log_{10}\alpha /N$, where α=0.05, and N=46,742). Moreover, the genomic inflation factor (λ) was calculated as the ratio of the median of the observed chi-square test statistics and the median of the expected chi-square distribution under the null hypothesis (df = 1). Finally, Manhattan and QQ plots were visualized using the CMplot package of R software (version 4.4.3) based on the GWAS results.

### 2.6. Functional Annotation

To figure out the potential function, annotation files were downloaded from the PigQTLdb website (release 57) [24] and intersected with these significant loci using the BEDtools software (version 2.31.1) [25]. Additionally, the significant loci identified in GWAS were also mapped to the pig reference genome (*Sus scrofa 11.1*) to find key candidate genes associated with teat number traits. In addition, the functions of these genes were annotated using the iSwine website [26] and DAVID website [27] (accessed on 6 April 2026). Finally, a phenome-wide association study (pheWAS) was conducted to find the association between these genes and reproduction traits using the PigBiobank website [28] (accessed on 10 April 2026).

## 3. Results and Discussion

### 3.1. Descriptive Statistics and Heritability of Teat Number Traits

**Figure 1:**
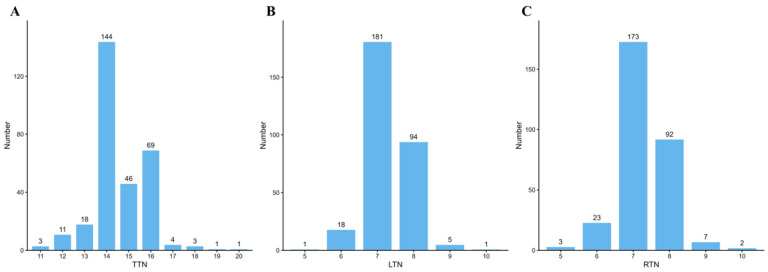
Frequency distribution of phenotype data. (**A**) Frequency distribution of total teat number (TTN) in study population. (**B**) Frequency distribution of left teat number (LTN) in study population. (**C**) Frequency distribution of right teat number (RTN) in study population.

In this study, the phenotypes of 300 Jishen Black sows were collected and analyzed. The descriptive statistics and heritability of TTN, LTN, and RTN were shown in Table 1. The ranges of TTN, LTN, and RTN were 11–20, 5–10, and 5–10, with mean values of 14.57, 7.29, and 7.28, respectively. The coefficient of variation (CV) of these teat number traits ranged from 8% to 10%, representing the relatively good consistency in our experimental population. According to Figure 1A, most JSB sows exhibited 14, 15, or 16 TTN (259, 86%). Only 41 pigs (14%) had TTN values outside this range. Interestingly, LTN and TTN exhibited a similar frequency distribution in the studied population (Figure 1B,C).

Interestingly, the heritability (*h*^2^) estimates of TTN in JSB pigs (0.19) were similar to those in Yorkshire pigs (Table 1). For instance, Fang et al. reported that the *h*^2^ of TTN in Danish Yorkshire pigs was 0.17 [8]. Additionally, the TTN of French Yorkshire pigs also exhibited *h*^2^ as 0.17 in Lin et al.’s research [9]. Among three traits, the LTN exhibited the highest *h*^2^ (0.22), while the *h*^2^ estimate of the RTN was the lowest (0.14). The low-to-medium heritability of teat number traits indicated moderate genetic control in JSB sows, suggesting that genetic markers would be helpful in improving phenotypes in the breeding programs.

### 3.2. Results of Genome-Wide Association Studies

**Figure 2:**
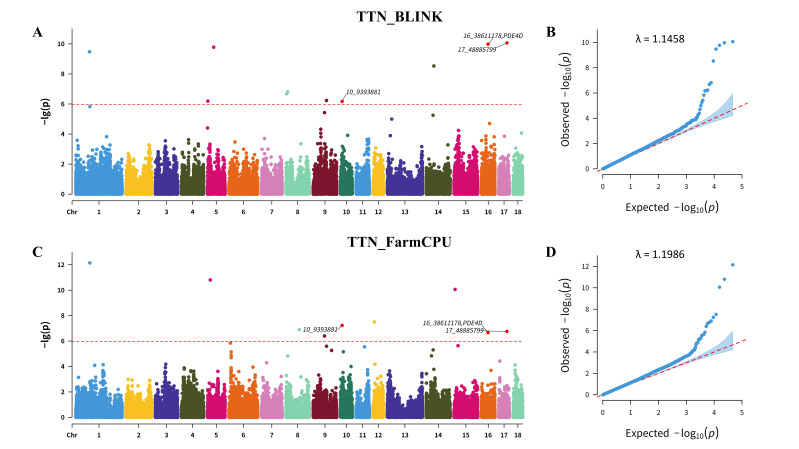
Manhattan and QQ plots of the total teat number (TTN). (**A**) Manhattan plot of the GWAS for TTN with the BLINK model. (**B**) QQ plot for TTN with the BLINK model. (**C**) Manhattan plot of the GWAS for TTN with the FarmCPU model. (**D**) QQ plot for TTN with the FarmCPU model. Different colors represent different chromosomes, and the red dashed line indicates the threshold. The threshold in the Manhattan plot was set as 5.971 (calculated as $-\log_{10}0.05/46,742$).

**Figure 3:**
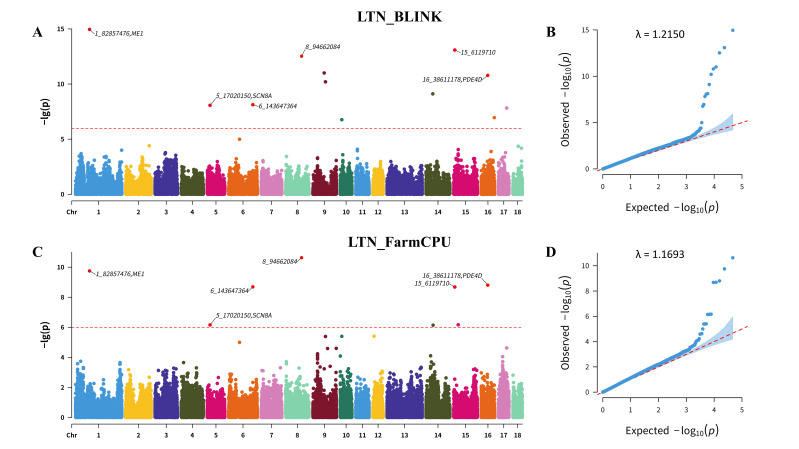
Manhattan and QQ plots of the left teat number (LTN). (**A**) Manhattan plot of the GWAS for LTN with the BLINK model. (**B**) QQ plot for LTN with the BLINK model. (**C**) Manhattan plot of the GWAS for LTN with the FarmCPU model. (**D**) QQ plot for LTN with the FarmCPU model. Different colors represent different chromosomes, and the red dashed line indicates the threshold. The threshold in the Manhattan plot was set as 5.971 (calculated as $-\log_{10}0.05/46,742$).

**Figure 4:**
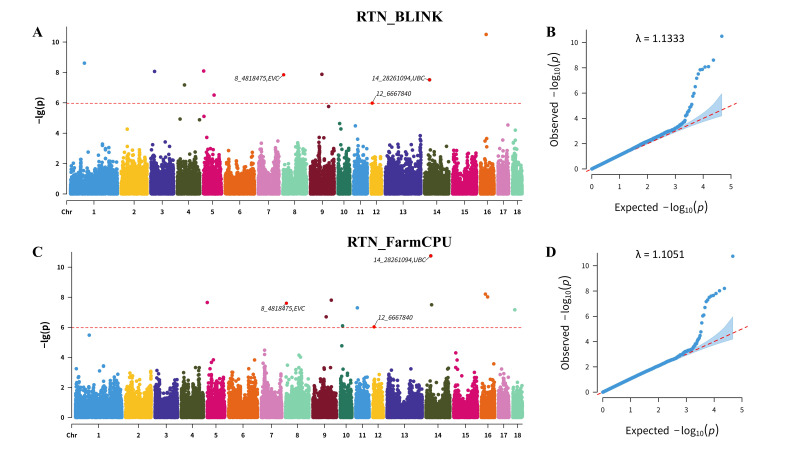
Manhattan and QQ plots of the right teat number (RTN). (**A**) Manhattan plot of the GWAS for RTN with the BLINK model. (**B**) QQ plot for RTN with the BLINK model. (**C**) Manhattan plot of the GWAS for RTN with the FarmCPU model. (**D**) QQ plot for RTN with the FarmCPU model. Different colors represent different chromosomes, and the red dashed line indicates the threshold. The threshold in the Manhattan plot was set as 5.971 (calculated as $-\log_{10}0.05/46,742$).

**Figure 5:**
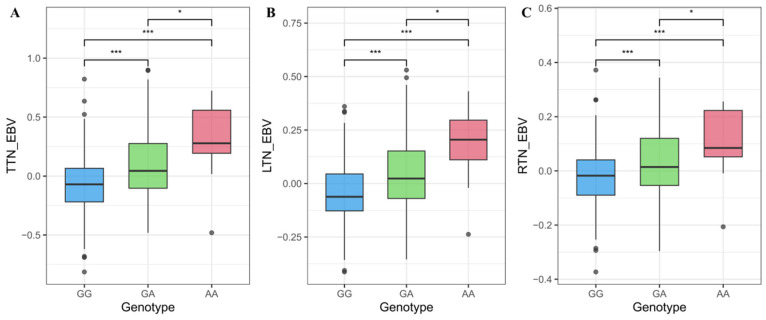
Box plots of estimated breeding values at the 16_38611178. (**A**) Box plot of estimated breeding values of total teat number (TTN_EBV). (**B**) Box plot of estimated breeding values of left teat number (LTN_EBV). (**C**) Box plot of estimated breeding values of right teat number (RTN_EBV). Significant differences between genotypes were determined by Welch’s *t*-test (* *p* < 0.05, *** *p* < 0.001).

The box plots of the estimated breeding values for the TTN, LTN, and RTN are shown in Figure S1. Additionally, Figure 2, Figure 3, Figure 4 and Figure 5 present the Manhattan and QQ plots of the TTN, LTN, and RTN based on the BLINK and FarmCPU models, respectively. The genomic inflation factor (λ) ranged from 1.11 to 1.22 across traits, indicating a mild inflation of test statistics (Figure 2B,D, Figure 3B,D and Figure 4B,D). This is commonly observed in pig GWAS due to complex familial relationships and population stratification. To mitigate potential false positives, we employed multi-locus models (BLINK and FarmCPU) that inherently account for background genetic variation through iterative cofactor selection and kinship matrix integration. Furthermore, only SNPs simultaneously significant in both models and passing the conservative Bonferroni threshold (*p* < 0.05/46,742) were retained as key loci, thereby ensuring robust association signals despite mild genomic inflation. In total, 40 significant SNPs were detected by genome-wide association studies and passed multiple testing correction (Table S1). Among them, 29 and 24 SNPs were significant based on the BLINK and FarmCPU models, respectively. Only the SNPs identified as significant by both the BLINK and FarmCPU models for the same trait were defined as key loci and mapped to the pig reference genome (*Sus Scrofa 11.1*) to detect key genes.

As shown in Figures S2A–C, S3 and S6, and three key loci were identified in the GWAS of TTN, LTN, and RTN, respectively, which are highlighted in the Manhattan plots (Figure 2A,C, Figure 3A,C and Figure 4A,C). Using the PigQTLdb website (release 57), only two key loci colocalized with existing quantitative trait loci (QTLs) (Table S2), including subcutaneous fat thickness [29], the arachidic acid to stearic acid ratio, and the Eicosenoic acid to eicosanoic acid ratio [30]. However, there was no QTL associated with reproduction traits, which highlighted the meaning of key significant SNPs found in this research.

Mapped to the reference genome, five key genes were detected, including malic enzyme 1 (*ME1*), sodium voltage-gated channel alpha subunit 8 (*SCN8A*), EvC ciliary complex subunit 1 (*EVC*), ubiquitin C (*UBC*), and phosphodiesterase 4D (*PDE4D*) (Table 2). Interestingly, variant 16_38611178 (rs81458863) was genome-wide significant in GWAS for both TTN and LTN, located in the region of *PDE4D*. The relationship between estimated breeding value (EBV) and the genotype of variant 16_38611178 is shown in Figure 5A–C. For this SNP, A is the dominant allele. The higher the frequency of allele A, the higher the EBV for the teat number traits in JSB sows. In a previous study, a QTL flanked by markers SW403 and SWR2086, which spans the *PDE4D* gene region, was found to be associated with nonfunctional nipples in a White Meishan × Duroc F2 resource population [31]. Additionally, a QTL encompassing the *ME1* gene region and associated with teat number traits was detected by Ding et al. in a White Duroc × Erhualian pig crossbred population [32].

To figure out the potential function of these key genes, gene ontology (GO) and Kyoto encyclopedia of genes and genomes (KEGG) annotation were conducted in this research (Tables S3 and S4). The *EVC* was annotated to two cellular components (ciliary basal body and ciliary membrane) and two biological processes (smoothened signaling pathway and its positive regulation) (Table S3). These GO terms are involved in mammary epithelial development and branching morphogenesis [33,34,35], potentially contributing to teat formation through regulating epithelial proliferation. In addition, the *ME1* and *UBC* were associated with the PPAR signaling pathway (Table S4). The PPAR signaling pathway can regulate tissue differentiation during embryonic development and modulate mammary lipid synthesis after birth [36,37]. Unfortunately, these terms cannot directly demonstrate their influence on teat formation. However, the smoothened signaling pathway and PPAR signaling pathway exhibit cooperative interactions with the Wnt signaling pathway [38,39], which has been demonstrated to affect embryonic teat formation [40,41].

Finally, we conducted a phenome-wide association study (pheWAS) using the PigBioBank website (accessed on 10 April 2026). The top 20 reproduction traits associated with *ME1*, *SCN8A*, *EVC*, *UBC*, and *PDE4D* were displayed in Figures S3–S7, respectively. Surprisingly, these key candidate genes exhibited high correlation with teat number traits. For instance, Y_TNUM and M_TNUM were the top two significant reproduction traits for *SCN8A*, *EVC*, and *PDE4D*. In summary, our findings provided 11 efficient molecular markers and five key genes to improve the teat number performance of JSB sows.

### 3.3. Limitation and Implication

In this study, we conducted a genome-wide association study with teat number traits in 300 matured Jishen Black sows. It is acknowledged that a sample size of 300 individuals is relatively small for GWAS analysis, which inevitably limits the statistical power to detect genetic variants with small or moderate effects, and increases the risk of both false-positive and false-negative associations. To mitigate these limitations, we employed multi-locus models (BLINK and FarmCPU), which are robust to small sample sizes by accounting for background genetic variation via iterative cofactor selection and kinship matrix integration. Additionally, the stringent Bonferroni correction and cross-model validation further reduced false-positive associations. Nevertheless, we recognize that our findings are primarily limited to identifying major-effect loci rather than capturing the full genetic architecture of teat number traits. Future studies with larger sample sizes, combined with multi-breed meta-analyses, are necessary to validate the detected associations, improve statistical power, and uncover additional minor-effect QTLs.

As a newly certified pig breed, Jishen Black pigs represent a synthetic line developed through the crossbreeding of commercial pig breeds with Chinese indigenous pigs. This unique genetic background endows the breed with abundant genetic diversity, making it an ideal and valuable resource for dissecting the genetic basis of complex quantitative traits, such as teat number. Despite its economic importance for sow prolificacy and lactation capacity, the genetic architecture underlying teat number variation in Jishen Black pigs remains largely unexplored, with very few systematic genetic studies reported to date. Therefore, the present study conducted the first comprehensive genome-wide association study to unravel the genetic mechanism governing teat number traits in Jishen Black pigs. Notably, unlike most previous GWAS that directly use raw teat counts, we employed EBVs estimated with contemporary group as a fixed effect as the phenotypic variable. Additionally, the first three principal components (PCs) were included as fixed factors in the GWAS model to further adjust for the population structure and polygenic background. Since the teat number is determined during early embryonic development and is unaffected by postnatal environmental factors [7], these adjustments effectively reduce population stratification noise and improve the reliability of marker-trait associations. Importantly, our analysis identified two novel candidate genes, *SCN8A* and *EVC*, as well as a shared pleiotropic locus encompassing *PDE4D*, which have not been widely documented or functionally validated in previous GWAS of teat number in pigs. These findings not only enrich our understanding of the genetic regulation of teat number development but also provide novel molecular markers and biological insights into the genetic architecture of teat number traits, laying a solid foundation for future molecular-assisted selection and genetic improvement in Jishen Black pigs and other pig populations.

## 4. Conclusions

In conclusion, this study represents the first genome-wide association analysis of teat number traits in the Jishen Black pig, a newly certified Chinese indigenous breed. By employing multi-locus GWAS models (BLINK and FarmCPU) in 300 JSB sows, we identified 11 significant SNPs and five key genes (*ME1*, *SCN8A*, *EVC*, *UBC*, and *PDE4D*) associated with total, left, and right teat numbers. These findings hold substantial practical implications for pig breeding and conservation. The significant SNPs provide reliable molecular markers that can be integrated into marker-assisted selection schemes. Furthermore, these markers establish a foundational resource for implementing genomic selection in the JSB pig population, which could substantially improve the accuracy of estimated breeding values and accelerate genetic gain for this economically important, sex-limited trait.

Collectively, our findings represent the first GWAS report for teat number in Jishen Black pigs, and provide novel candidate genes and molecular markers that complement existing knowledge on the genetic basis of teat number traits in pigs.

## Figures and Tables

**Table 1:** Descriptive statistics and heritability of teat number traits.

Trait	Number	Mean ± SD	Max	Min	CV (%)	*h*^2^ ± SE
TTN	300	14.57 ± 1.24	20	11	8.51	0.19 ± 0.12
LTN	300	7.29 ± 0.63	10	5	8.64	0.22 ± 0.12
RTN	300	7.28 ± 0.71	10	5	9.75	0.14 ± 0.11

TTN: total teat number; LTN: left teat number; RTN: right teat number; SD: standard deviation; CV: coefficient of variation; *h*^2^: heritability; SE: standard error.

**Table 2:** Details of key candidate loci and genes.

SNP	Chromosome	Position	Trait	Gene
10_9393881	10	9393881	TTN	-
16_38611178	16	38611178	TTN	*PDE4D*
17_48885799	17	48885799	TTN	-
1_82857476	1	82857476	LTN	*ME1*
5_17020150	5	17020150	LTN	*SCN8A*
6_143647364	6	143647364	LTN	-
8_94662084	8	94662084	LTN	-
15_6119710	15	6119710	LTN	-
16_38611178	16	38611178	LTN	*PDE4D*
8_4818475	8	4818475	RTN	*EVC*
12_6667840	12	6667840	RTN	-
14_28261094	14	28261094	RTN	*UBC*

- indicates the SNP loci are not located in genes.

## Data Availability

The original contributions presented in this study are included in the article/Supplementary Material. Further inquiries can be directed to the corresponding author(s).

## Supplementary Materials

The following supporting information can be downloaded at https://www.mdpi.com/article/10.3390/vetsci13060537/s1, Table S1: Details of all candidate loci and genes. Table S2: Quantitative trait loci mapping of key candidate loci. Table S3: Results of GO enrichment of key candidate loci. Table S4: Results of KEGG enrichment of key candidate loci. Figure S1: Violin plots of estimated breeding values. Figure S2: Venn diagrams of candidate loci identified for teat number traits. Figure S3: Top 20 reproduction traits of pheWAS for *ME1*. Figure S4: Top 20 reproduction traits of pheWAS for *SCN8A*. Figure S5: Top 20 reproduction traits of pheWAS for *EVC*. Figure S6: Top 20 reproduction traits of pheWAS for *UBC*. Figure S7: Top 20 reproduction traits of pheWAS for *PDE4D*.

## Abbreviations

The following abbreviations are used in this manuscript:

TTN	Total Teat Number
LTN	Left Teat Number
RTN	Right Teat Number
JSB	Jishen Black
GWAS	Genome-Wide Association Study
SNP	Single Nucleotide Polymorphism
EBV	Estimated Breeding Value
BLINK	Bayesian-information and Linkage-disequilibrium Iterated Conditional Key
FarmCPU	Fixed and random model Circulating Probability Unification
QTL	Quantitative Traits Locus
GO	Gene Ontology
KEGG	Kyoto Encyclopedia of Genes and Genomes
pheWAS	Phenome-Wide Association Study
