# Supplementary material for: Genome-Wide Association Study Revealed Key Genes with the Teat Number in Jishen Black Pigs
Source: Vet Sci. 2026 May 29;13(6):537. doi: 10.3390/vetsci13060537 (PMC13307678; doi:10.3390/vetsci13060537)
Supplement: Supplementary file 1 [file vetsci-13-00537-s001.zip › Supplementary materials.pdf]

Table S1. Details of all candidate loci and genes.

SNP	Chromosome	Position	Trait	Gene
1_81033340	1	81033340	BLINK.TTN_EBV	-
1_81162933	1	81162933	BLINK.RTN_EBV	-
			FarmCPU.TTN_EBV	
1_82857476	1	82857476	BLINK.LTN_EBV	*ME1*
			FarmCPU.LTN_EBV	
3_21604913	3	21604913	BLINK.RTN_EBV	-
4_41745551	4	41745551	BLINK.RTN_EBV	-
5_697684	5	697684	FarmCPU.RTN_EBV	-
5_784088	5	784088	BLINK.RTN_EBV	*BRD1*
5_2413655	5	2413655	BLINK.TTN_EBV	-
			FarmCPU.TTN_EBV	
5_17020150	5	17020150	BLINK.LTN_EBV	*SCN8A*
			FarmCPU.LTN_EBV	
5_36953856	5	36953856	BLINK.TTN_EBV	-
5_61759160	5	61759160	BLINK.RTN_EBV	*GABARAPL1*
			BLINK.LTN_EBV	
6_143647364	6	143647364		-
			FarmCPU.LTN_EBV	
8_4818475	8	4818475	BLINK.TTN_EBV	*EVC*

			BLINK.RTN_EBV<br>FarmCPU.RTN_EBV	
8_10871768	8	10871768	BLINK.TTN_EBV	*CC2D2A*
8_79158996	8	79158996	FarmCPU.TTN_EBV	*IQCM*
8_94662084	8	94662084	BLINK.LTN_EBV<br>FarmCPU.LTN_EBV	-
9_67032967	9	67032967	FarmCPU.TTN_EBV<br>BLINK.LTN_EBV	-
9_67389371	9	67389371	BLINK.RTN_EBV	-
9_74519626	9	74519626	BLINK.LTN_EBV	-
9_78754343	9	78754343	FarmCPU.RTN_EBV	*NXPH1*
9_78948675	9	78948675	BLINK.TTN_EBV	*NXPH1*
9_109099580	9	109099580	FarmCPU.RTN_EBV	-
10_9393881	10	9393881	FarmCPU.TTN_EBV<br>BLINK.LTN_EBV	-
10_13938246	10	13938246	BLINK.TTN_EBV<br>FarmCPU.RTN_EBV	*H3-3A*
11_9541267	11	9541267	FarmCPU.RTN_EBV	-
12_6667840	12	6667840	FarmCPU.TTN_EBV<br>BLINK.RTN_EBV<br>FarmCPU.RTN_EBV	-

14_28261094	14	28261094	BLINK.RTN_EBV<br>FarmCPU.RTN_EBV	*UBC*
14_33124700	14	33124700	FarmCPU.RTN_EBV	*CIT*
14_40162411	14	40162411	BLINK.LTN_EBV	*GCN1*
14_41862687	14	41862687	FarmCPU.LTN_EBV	*SVOP*
14_44633923	14	44633923	BLINK.TTN_EBV	-
			FarmCPU.TTN_EBV	
15_6119710	15	6119710	BLINK.LTN_EBV	-
			FarmCPU.LTN_EBV	
15_26381236	15	26381236	FarmCPU.LTN_EBV	-
16_24873353	16	24873353	FarmCPU.RTN_EBV	-
16_33008580	16	33008580	BLINK.RTN_EBV	-
16_37895943	16	37895943	FarmCPU.RTN_EBV	-
16_38611178	16	38611178	BLINK.TTN_EBV<br>FarmCPU.TTN_EBV<br>BLINK.LTN_EBV<br>FarmCPU.LTN_EBV	*PDE4D*
16_77515310	16	77515310	BLINK.LTN_EBV	-
			BLINK.TTN_EBV	
17_48885799	17	48885799	FarmCPU.TTN_EBV	-
			BLINK.LTN_EBV	

18_11312592	18	11312592	FarmCPU.RTN_EBV	-

- indicates the SNP loci is not located in genes.

Table S2. Quantitative trait loci mapping of key candidate loci.

SNP	Trait	QTL_ID	Name	PUBMED_ID
8_94662084	Meat_and_Carcass_Association	139240	Subcutaneous fat thickness	28831160
16_38611178	Meat_and_Carcass_Association	101582	Arachidic acid to stearic acid ratio	27097669
16_38611178	Meat_and_Carcass_Association	101720	Eicosenoic acid to eicosanoic acid ratio	27097669

Table S3. Results of GO enrichment of key candidate loci.

Gene	Term	Type
ME1	pyruvate metabolic process	biological process
	malate metabolic process	biological process
	NADP+ metabolic process	biological process
	response to hormone	biological process
	protein homotetramerization	biological process
	regulation of NADP metabolic process	biological process
	cytoplasm	cellular component
	mitochondrion	cellular component
	cytosol	cellular component
	magnesium ion binding	molecular function

	malic enzyme activity	molecular function
	malate dehydrogenase (decarboxylating) (NAD+) activity	molecular function
	malate dehydrogenase (decarboxylating) (NADP+) activity	molecular function
	oxaloacetate decarboxylase activity	molecular function
	manganese ion binding	molecular function
	identical protein binding	molecular function
	metal ion binding	molecular function
	NAD binding	molecular function
SCN8A	action potential	biological process
	neuronal action potential	biological process
	sensory perception of pain	biological process
	monoatomic ion transmembrane transport	biological process

	sodium ion transmembrane transport	biological process
	membrane depolarization during action potential	biological process
	voltage-gated sodium channel complex	cellular component
	plasma membrane	cellular component
	cell junction	cellular component
	axon	cellular component
	voltage-gated sodium channel activity	molecular function
	protein binding	molecular function
	sodium ion binding	molecular function
	endochondral bone growth	biological process
EVC	smoothened signaling pathway	biological process
	positive regulation of smoothened signaling pathway	biological process

	cytoplasm	cellular component
	cytoskeleton	cellular component
	ciliary basal body	cellular component
	ciliary membrane	cellular component
	plasma membrane protein complex	cellular component
	protein ubiquitination	biological process
	modification-dependent protein catabolic process	biological process
	nucleus	cellular component
UBC	cytoplasm	cellular component
	mitochondrial outer membrane	cellular component
	protein tag activity	molecular function
	ubiquitin protein ligase binding	molecular function

PDE4D	cAMP catabolic process	biological process
	signal transduction	biological process
	positive regulation of type II interferon production	biological process
	positive regulation of interleukin-2 production	biological process
	positive regulation of interleukin-5 production	biological process
	T cell receptor signaling pathway	biological process
	establishment of endothelial barrier	biological process
	regulation of cardiac muscle cell contraction	biological process
	negative regulation of adenylate cyclase-activating G protein-coupled receptor signaling pathway	biological process
	negative regulation of cAMP/PKA signal transduction	biological process
	regulation of cell communication by electrical coupling involved in cardiac conduction	biological process
	nucleus	cellular component

nucleoplasm	cellular component
cytosol	cellular component
plasma membrane	cellular component
cilium	cellular component
nuclear membrane	cellular component
calcium channel complex	cellular component
perinuclear region of cytoplasm	cellular component
3',5'-cyclic-nucleotide phosphodiesterase activity	molecular function
3',5'-cyclic-AMP phosphodiesterase activity	molecular function
cAMP binding	molecular function
transmembrane transporter binding	molecular function
metal ion binding	molecular function

	3',5'-cyclic-GMP phosphodiesterase activity	molecular function
	ATPase binding	molecular function
	scaffold protein binding	molecular function

Table S4. Results of KEGG enrichment of key candidate loci.

Gene	Term
ME1	Pyruvate metabolism
	Metabolic pathways
	Carbon metabolism
	PPAR signaling pathway
SCN8A	IgSF CAM signaling
EVC	Hedgehog signaling pathway
UBC	PPAR signaling pathway
	Ubiquitin mediated proteolysis
	Mitophagy - animal
	Autophagy - animal
	Parkinson disease
	Pathways of neurodegeneration - multiple diseases
	Kaposi sarcoma-associated herpesvirus infection
PDE4D	Purine metabolism
	Metabolic pathways
	cAMP signaling pathway
	Parathyroid hormone synthesis, secretion and action
	Morphine addiction

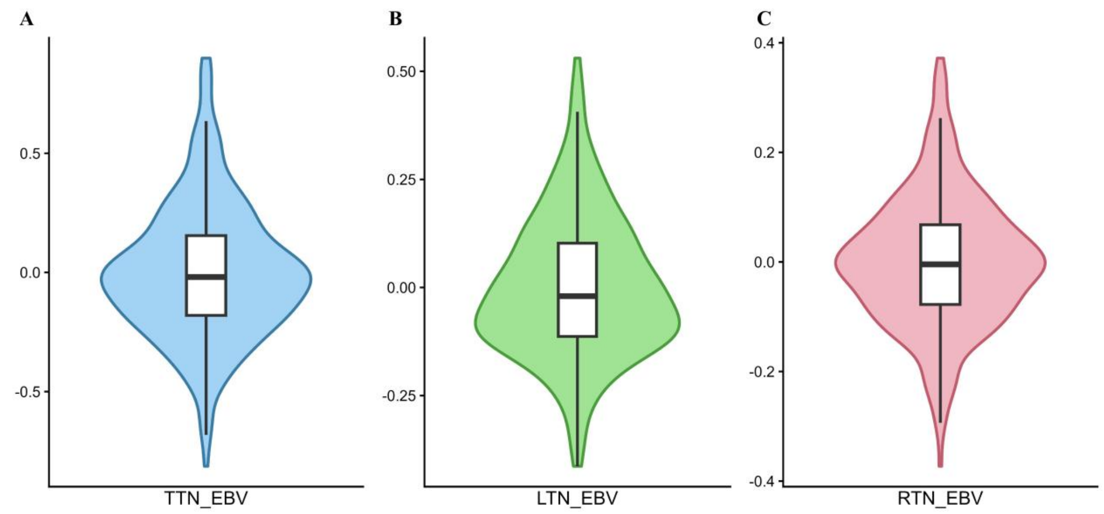

Figure S1. Violin plots of estimated breeding values. (A) Violin plot of estimated breeding values of total teat number (TTN_EBV). (B) Violin plot of estimated breeding values of left teat number (LTN_EBV). (C) Violin plot of estimated breeding values of right teat number (RTN_EBV).

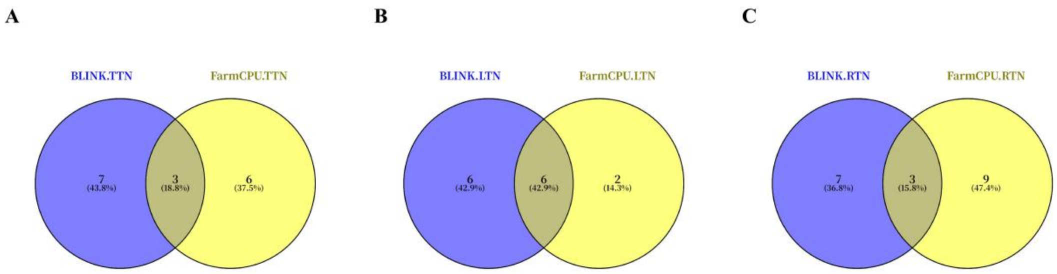

Figure S2. Venn diagrams of candidate loci identified for teat number traits. (A) Venn diagram of candidate loci identified for total teat number (TTN). (B) Venn diagram of candidate loci identified for left teat number (LTN). (A) Venn diagram of candidate loci identified for right teat number (RTN).

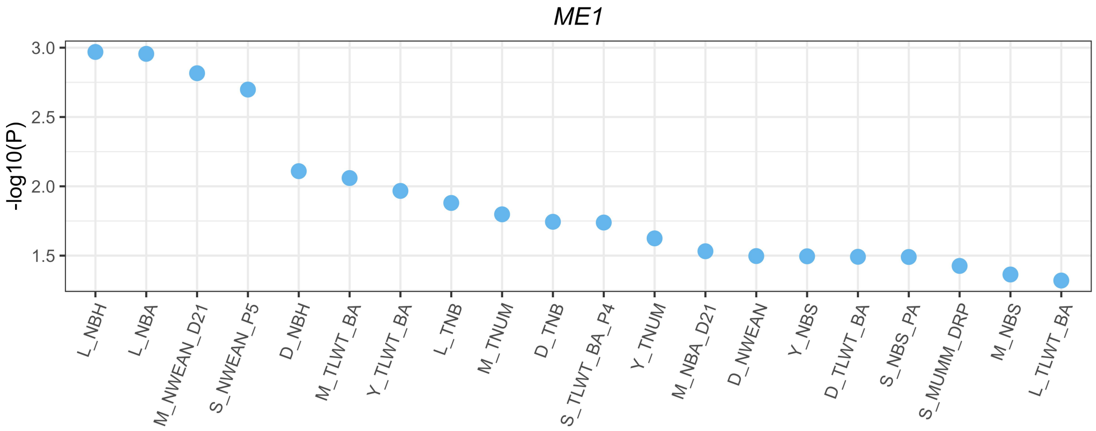

Figure S3. Top 20 reproduction traits of pheWAS for *ME1*.

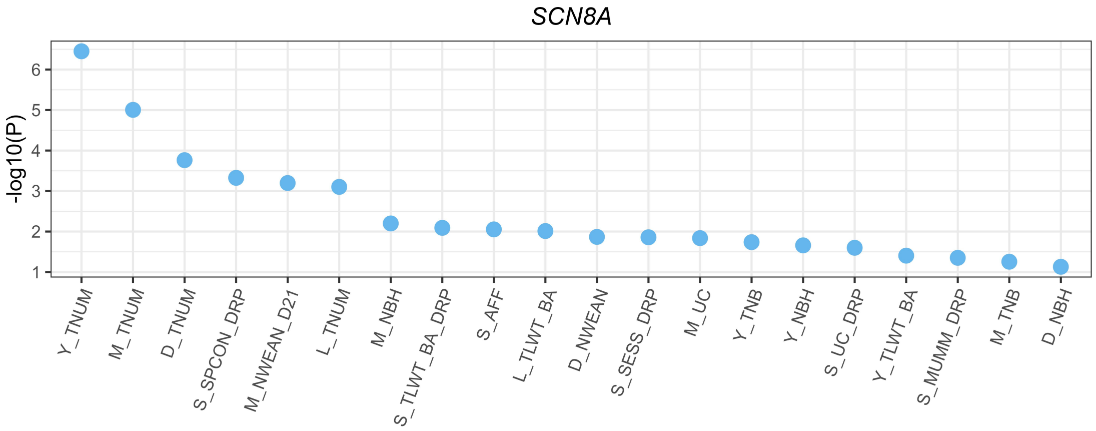

Figure S4. Top 20 reproduction traits of pheWAS for *SCN8A*.

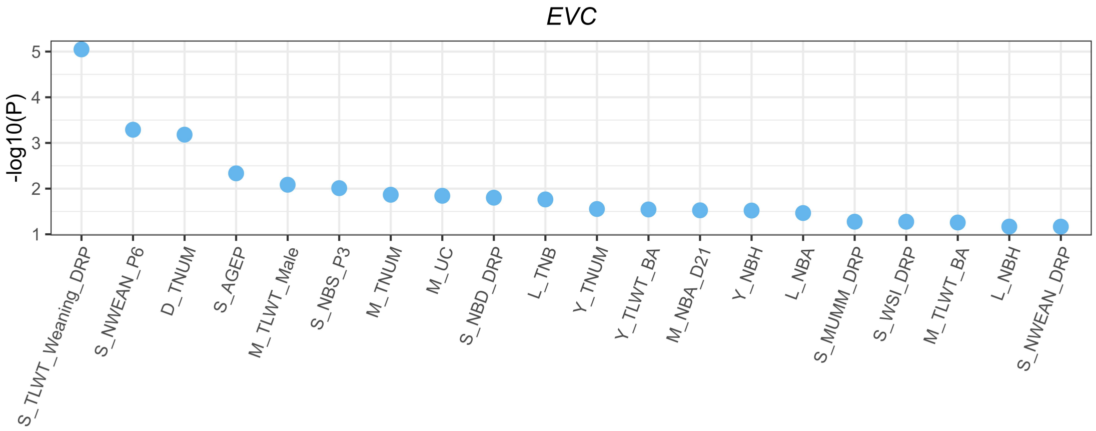

Figure S5. Top 20 reproduction traits of pheWAS for *EVC*.

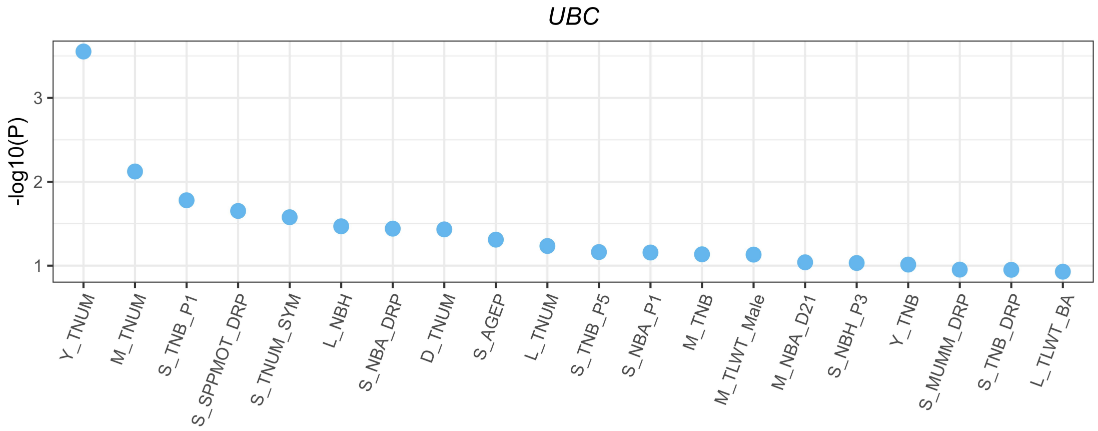

Figure S6. Top 20 reproduction traits of pheWAS for *UBC*.

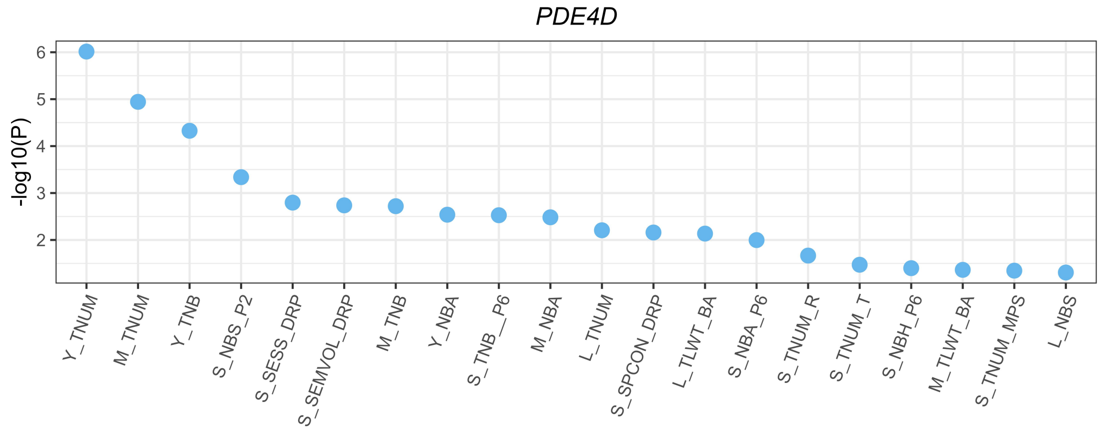

Figure S7. Top 20 reproduction traits of pheWAS for *PDE4D*.
